# Unveiling the Spectrum: Clinical and Molecular Insights from a Spanish Pediatric Cohort with Hypermobility Disorders and Ehlers-Danlos Syndrome

**DOI:** 10.3390/genes16080925

**Published:** 2025-07-31

**Authors:** David Foz Felipe, Dídac Casas-Alba, Sara H. Sadok, Marina Toral Fernández, Lourdes Vega-Hanna, Laura Plaza, Asunción Vicente Villa, Judith Armstrong, Encarna Guillén-Navarro, Antonio F. Martínez-Monseny

**Affiliations:** 1 Pediatrics Department, Hospital Sant Joan de Déu, 08950 Barcelona, Spain; david.foz@sjd.es (D.F.F.); marina.toral@sjd.es (M.T.F.); laura.plaza@sjd.es (L.P.); 2 Department of Medical Genetics, Hospital Sant Joan de Déu, Institut de Recerca Sant Joan de Déu, 08950 Barcelona, Spain; didac.casas@sjd.es (D.C.-A.); sara.hadj@sjd.es (S.H.S.); encarna.guillen@sjd.es (E.G.-N.); 3 Department of Genetics, Hospital Santa Creu i Sant Pau, 08950 Barcelona, Spain; lourdes.vega@sjd.es; 4 Department of Dermatology, Hospital Sant Joan de Déu, 08950 Barcelona, Spain; asuncion.vicente@sjd.es; 5 Genomic Unit, Department of Genetic and Molecular Medicine, Hospital Sant Joan de Déu, Institut de Recerca Sant Joan de Déu, 08950 Barcelona, Spain; judith.armstrong@sjd.es; 6 CIBERER, Centro de Investigación Biomédica en Red de Enfermedades Raras, 28029 Madrid, Spain

**Keywords:** Ehlers-Danlos syndrome, hypermobility spectrum disorders, joint hypermobility, collagenopathy, pediatrics

## Abstract

Diagnosing hypermobility disorders and Ehlers-Danlos syndrome (EDS) in children is challenging due to overlapping features with generalized joint hypermobility (GJH) and the lack of biomarkers. **Background/Objectives**: This study aims to describe the clinical and genetic features of pediatric EDS patients and identify key comorbidities and correlations. **Methods**: This is a single-center observational study of patients under 18 diagnosed with suspicion of EDS (2018–2024) at a tertiary pediatric hospital. Diagnoses were made using 2017 criteria. **Results**: Forty-one patients (46% female; mean age 11.1 ± 2.8 years) were included. Based on 2017 criteria, 61% had hypermobile EDS (hEDS)/hypermobility spectrum disorder (HSD), 22% classical EDS, 7.3% vascular, and 9.7% other subtypes. Musculoskeletal (90.2%), cutaneous (68.3%), and psychiatric (56.1%) symptoms were most frequent. Significant associations included older age with psychiatric symptoms (*p* = 0.029), Beighton score with dislocations (*p* = 0.026), and less atrophic scarring in hEDS (*p* < 0.008). Genetic testing (73% performed) confirmed pathogenic variants (11 novel) in EDS with a known molecular cause. **Conclusions**: This study proposes a clinically guided approach and diagnostic algorithm for youth hypermobility, emphasizing precision medicine principles, while highlighting the urgent need for further research to identify hEDS biomarkers.

## 1. Introduction

Ehlers-Danlos Syndrome (EDS) encompasses a group of inherited disorders that affect connective tissue, primarily due to defects in the structure, synthesis, or processing of collagen, or in proteins that interact with collagen. These defects typically lead to manifestations such as increased joint hypermobility, skin hyperextensibility, and tissue fragility. 

The classification of EDS, particularly the hypermobile subtype (hEDS), has posed a clinical challenge in pediatric populations for many years. Generalized joint hypermobility (GJH) is relatively common in children and adolescents and can be considered a physiological trait in many cases. It is often a normal developmental feature in younger individuals, as their joints tend to be more flexible during growth. However, the persistence of GJH, particularly when accompanied by symptoms such as pain or joint instability, may raise suspicion for an underlying connective tissue disorder like EDS. In this context, when dealing with syndromic hypermobility, it is crucial for the clinician to take into consideration the potential burden of premature labeling and overdiagnosis, given their significant clinical and psychosocial implications. However, it is also important to maintain awareness and clinical suspicion, which may help prevent underdiagnosis.

The 2017 international EDS classification defined 13 subtypes, all with known molecular basis except hypermobile EDS (hEDS), for which newer clinical criteria were introduced due to the lack of a genetic marker [1]. These criteria have served as the diagnostic standard for both adults and children. Nevertheless, applying adult-based criteria to pediatric populations is challenging, as children may not yet exhibit a definitive phenotype. This challenge is further complicated by the fact that certain features and comorbidities associated with EDS are more common in childhood and may occur transiently without resulting in a definitive diagnosis [2].

Additionally, the 2017 framework introduced the concept of Hypermobility Spectrum Disorders (HSDs) to describe individuals with symptomatic joint hypermobility who do not fully meet the hEDS criteria [2].

In response to these limitations, recently, a 2023 framework by Tofts et al. proposed a pediatric-specific classification aimed at improving the evaluation of GJH in children before biological maturity [2]. Although promising, this classification is still under early clinical implementation and has not been universally adopted in practice.

The significant clinical and genetic heterogeneity of HSD and EDS, particularly in children, coupled with their overlapping characteristics with other connective tissue disorders, frequently leads to errors and delays in diagnosis [3,4,5,6,7]. Some of the main symptoms in EDS and HSD include GJH and skin and tissue abnormalities. These may be accompanied by acute or chronic musculoskeletal complications and other comorbidities [1,7,8,9,10], leading to a significant impact on quality of life.

The true incidence of EDS is likely underestimated, particularly in individuals with mild, atypical, or overlapping presentations [11]. In such cases, where clinical features are non-specific or diverge from the classical phenotype, genetic testing is essential for confirming molecular subtypes and excluding phenocopies. Accurate diagnosis requires expert clinical assessment followed by targeted genetic analysis [4].

In this study, we present a cohort of children and adolescents diagnosed with HSD and EDS based on the 2017 international classification. Our objective is to assess and describe their characteristics and comorbidities, enhancing current knowledge regarding clinical presentation and diagnostic complexity in the pediatric population.

## 2. Materials and Methods

A single-center prospective and retrospective observational study was carried out at a tertiary pediatric hospital. This study was approved by the Research and Ethics Committee of Hospital Sant Joan de Déu, Barcelona, Spain. The study was conducted in accordance with the Declaration of Helsinki, Good Clinical Practices, and applicable regulatory requirements. All parents and adult patients provided written informed consent, and adolescent patients able to understand the procedure gave their assent prior to patient enrollment.

Patients were initially referred to the Medical Genetics Department by general pediatricians or pediatric subspecialists for evaluation of suspected connective tissue disorders. The most common reasons for referral to the Medical Genetics Department included GJH with presence of chronic joint pain, especially in patients with hEDS or HSD. In other subtypes with typically later-onset features, such as vascular EDS, referral was often prompted by the identification of a pathogenic variant in a first-degree relative. In some cases, geneticists also initiated referral when a suggestive phenotype was identified in a relative. When a clinical suspicion or diagnostic confirmation was made, patients were included in our database. Data were obtained by reviewing digitalized medical records.

All patients under 18 years of age diagnosed with EDS and HSD between the years 2018 and 2024 were included in the study. Diagnoses were assigned according to the 2017 international classification criteria.

Patients with incomplete information in their medical history, and aged over 18 years at diagnosis, were excluded. Those with an alternative diagnosis after genetic testing were also excluded.

### 2.1. Measures

For each patient, data regarding age, sex, familial history, prenatal and perinatal history, and clinical manifestations were included. These manifestations were grouped into several categories including traumatological and musculoskeletal, neurological, psychiatric, gastrointestinal, ophthalmologic, cardiovascular and dermatologic. Hypermobility was measured using the Beighton criteria [12]. The Beighton score, and most clinical manifestations, were assessed by clinical geneticists. Although the initial Beighton score was sometimes assessed by general pediatricians to guide referral, all patients included in the study were later evaluated by experienced clinical geneticists, who confirmed the Beighton score using standardized criteria. The final diagnosis and inclusion in the study were based exclusively on the assessment performed by the Medical Genetics Department. In some specific cases where complementary exams were performed (e.g., echocardiography, neuroimaging), the diagnosis was made by pediatric subspecialists like cardiologists, neurologists, etc. Other manifestations, like dermatologic and psychiatric manifestations (e.g., cases of anxiety or depression that required pharmacologic treatment), were also evaluated by specific subspecialties. Although some of the mentioned clinical findings were confirmed by the corresponding subspecialists, when collaboration was required the final diagnostic decision of each patient was made collaboratively between the Medical Genetics Department and the respective subspecialty teams.

Genetic findings were included, when present, as well as whether genetic testing had been carried out in parents or first-degree relatives.

EDS subtypes were classified by clinical and molecular findings, following the 2017 criteria [1].

### 2.2. Molecular Studies

Genetic testing was considered in patients with joint hypermobility when there were additional clinical features suggestive of a connective tissue disorder such as abnormal skin findings, vascular anomalies, marked musculoskeletal complications, or a family history of connective tissue disorders. Testing was also conducted when clinical suspicion existed for a specific EDS subtype with known molecular basis

Genetic testing was performed by next-generation-sequencing (NGS). Clinical exome sequencing tests were performed at the Genetics Laboratory in Sant Joan de Déu Hospital (with Template Switch Oligo and Agilent Next Generation Sequencing). Results were reported by molecular geneticists. Variant analysis and interpretation were performed on genes with biological relevance in collagenopathies. Variants were classified using the American College of Medical Genetics and Genomics guidelines [13].

### 2.3. Statistical Analyses

Quantitative variables were reported as the mean (standard deviation). Qualitative variables were described using percentages. The Mann–Whitney U test was used to compare variables with nonparametric distribution in both groups. SPSS 21.00 (IBM Corp.; Armonk, NY, USA) was used for statistical analysis. A *p*-value of *p* < 0.05 was considered to be statistically significant.

### 2.4. Use of Generative Artificial Intelligence (GenAI)

Figures 2 and 3 in this manuscript were created with the assistance of generative artificial intelligence using NAPKIN.AI (version 0.13.2). No generative AI tools were used for text generation or any other aspect of the manuscript preparation.

## 3. Results

### 3.1. Characteristics of the Sample

The study included 41 patients diagnosed with EDS. The mean age at diagnosis was 11.1 ± 2.8 years, with 85% belonging to the group between 6 and 15 years. In terms of sex distribution, 46% were female and 54% were male. The median Beighton score across the cohort was 7.

According to the 2017 international classification of EDS, and after excluding patients with an alternative diagnosis, 61% (25) of the pediatric patients in our cohort were diagnosed with either hEDS or HSD, the latter applied to individuals who did not fully meet the criteria for hEDS.

Classical EDS accounted for 22% of cases (including both patients with classical type 1 and type 2 EDS). Vascular EDS was observed in 7.3% of patients. Additionally, 1 patient was diagnosed with kyphoscoliotic EDS, 1 patient with dermatosparaxis subtype and 2 with arthrochalasia subtype.

Regarding familial history, 51.2% of the patients had affected first-degree relatives. The genetic study in parents demonstrated maternal inheritance in 13.2%, and almost 34% of the mothers had compatible symptoms without studies performed. The diagnosis of the relatives preceded the child’s diagnosis in 83.3% of the cases. Table 1 summarizes the distribution of EDS subtypes in our pediatric cohort based on the 2017 international classification.

### 3.2. Clinical Manifestations

In terms of clinical manifestations (Table 2), musculoskeletal symptoms were the most common complication (90.2%), with chronic joint pain being the most frequent feature (63.4%). Feet deformities were present in 41.5% of patients, pes planus being the most frequent. Meanwhile, bone fractures were relatively rare, occurring in only 4.9% of patients. A higher Beighton score was significantly associated with the presence of recurrent dislocations (*p* = 0.026).

Cutaneous manifestations were also observed in a considerable proportion of patients (up to 68.3%). The most frequent features included skin hyperextensibility and easy bruising, while atrophic or abnormal scarring was less frequent. Interestingly, atrophic scarring was significantly less frequent among patients with hypermobile EDS compared to other subtypes (*p* < 0.008). When grouped by subtype, the classical forms of EDS showed the highest cutaneous involvement, with 88.9% presenting manifestations. All patients with the vascular subtype also had skin manifestations, primarily in the form of hematomas.

Regarding psychiatric conditions, these were more commonly reported in GJH/hypermobile EDS patients, with 57.1% presenting symptoms, in contrast to 55.6% of patients with the classical subtype. Psychiatric comorbidities were also present in 50% of patients with vascular EDS. Anxiety and depression were the most noted features, while ASD (autism spectrum disorder) was the least frequent. Additionally, older age was significantly associated with the presence of psychiatric disorders overall (*p* = 0.029). When stratified by specific diagnoses, the associations were not statistically significant for ADHD (*p* = 0.297) and for anxiety and depression (*p* = 0.294).

Cardiovascular manifestations were observed in 26.8% of patients in the cohort, the most frequent being syncope in 17.1%. Structural cardiac abnormalities identified included mitral valve prolapse in three patients and mitral valve insufficiency in two. Only one case of postural orthostatic tachycardia syndrome (POTS) was described. No cases of aortic root dilation or other serious life-threatening conditions were identified.

Neurological manifestations were reported in 51.2% of patients, with migraine headaches being the most common finding. No cases of Arnold-Chiari malformation were identified in this series.

Digestive disorders were present in 41.5% of patients, with chronic abdominal pain being the most frequent (22%). Notably, chronic abdominal pain was significantly associated with the presence of migraines (*p* = 0.045). Six patients were diagnosed with hernias, including three patients with inguinal hernias, and the rest with umbilical, diaphragmatic, and epigastric hernias. Five patients reported food intolerances or allergies.

Finally, ophthalmological manifestations were present in 43.9% of all patients. The only abnormality detected was refractive defects.

Although not applied in this study, recent pediatric-specific classification proposals like the 2023 classification by Tofts et al. allows for grouping patients with symptomatic hypermobility who have not yet reached biological maturity according to their predominant symptoms [2]. Age-specific classifications like the one mentioned help facilitate patient-centered follow-up aligned with personalized precision medicine and avoid unnecessary monitoring, given the low prevalence of some comorbidities in this population.

### 3.3. Genetic Aspects

Clinical exome analysis was performed in 30 patients (73%), while the remaining individuals with hEDS/HSD were diagnosed based on clinical criteria alone. However, up to 56% of patients with hEDS/HSD did undergo a genetic study. The diagnostic yield in EDS subtypes with known genetics (74%) was 100%. No clear candidate variants were detected in 26% patients, all of them being clinically compatible with hEDS/HSD. The molecular results are summarized in Table 3. VUS found in hEDS/HSD patients are included in Supplementary Table S1.

## 4. Discussion

This study represents one of the largest comprehensive clinical and molecular characterizations of pediatric patients with EDS and HSD in Spain. By providing a detailed analysis of comorbidities and diagnostic challenges in this population, it offers a foundation for developing tailored management and follow-up strategies in pediatric care.

Previous international studies have largely investigated pediatric joint hypermobility and EDS separately, primarily using the 2017 classification criteria [14,15,16,17]. While these studies have advanced knowledge of musculoskeletal, gastrointestinal, orthopedic, and autonomic comorbidities, they often lack an integrated diagnostic framework. Although not applied to our cohort, the recent 2023 classification proposed by Tofts et al. introduces a new sensitive approach that may help refine diagnostic categorization in pediatric populations as they mature, by taking into account physiological hypermobility and flexible categories for classifying patients that can evolve as clinical features develop. This approach may reduce the risk of premature labeling, overdiagnosis, and the unnecessary use of diagnostic tests and healthcare resources, while improving diagnostic accuracy compared to the 2017 version. Its clinical use remains limited, but it provides a promising structure for future studies.

**Figure 1:**
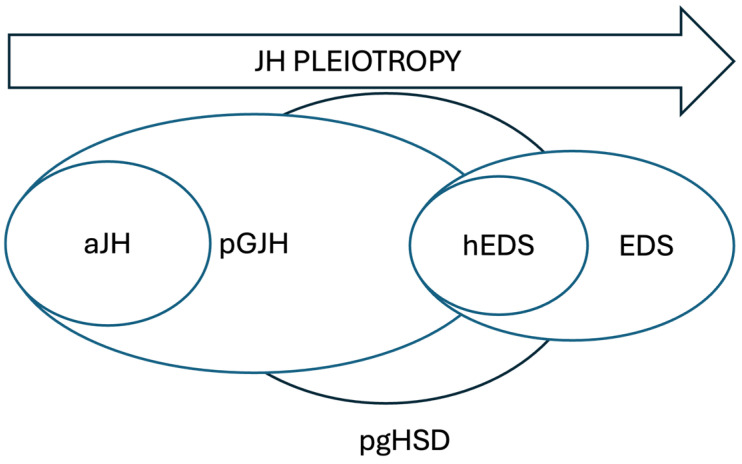
Diagram of the terminology with the revised new classification for Ehlers-Danlos syndrome (EDS) and joint hypermobility (JH) in the pediatric population. Pediatric generalized joint hypermobility (pGJH) and hypermobile EDS (hEDS) are closely related concepts, both falling within pediatric hypermobility spectrum disorders (pgHSDs). These disorders also include cases of asymptomatic joint hypermobility (aJH) and, at the other end, cases of EDS of genetic origin.

As a conceptual complement, we include a diagram that illustrates the evolving terminology and interactions within the joint hypermobility spectrum, including recent pediatric classifications not applied in this study (Figure 1).

Our study provides a comprehensive characterization of HSD/EDS manifestations in a pediatric cohort, highlighting both consistencies and novel observations compared to prior adult-focused research [4,10,13,18,19,20]. As expected, joint hypermobility with secondary chronic pain was predominant. Frequent musculoskeletal deformities, including pes planus, pectus abnormalities, and scoliosis, underscore early connective tissue fragility. Cutaneous signs such as skin hyperextensibility and bruising were common, while atrophic scarring was notably less frequent in hEDS, aiding differentiation from classical EDS.

Cardiovascular abnormalities were generally mild and non-life-threatening, with syncope cases rarely linked to structural defects, suggesting dysautonomia as a key factor. Ophthalmologic involvement was minimal [21]. Neurologically, headaches were the most prevalent complaint, reported in about one-third of patients, with multifactorial etiologies proposed [18,19]. Psychiatric comorbidities, particularly anxiety and depression, showed high prevalence and correlation with older age, reinforcing previous findings [22,23]. These significantly impact quality of life and highlight the importance of early mental health screening and tailored follow-up. Gastrointestinal symptoms such as recurrent abdominal pain and constipation were common, consistent with earlier reports [5,17,20,24]. Notably, we identified a significant association between chronic abdominal pain and migraine headaches. After excluding organic causes, we propose systemic dysautonomia and psychosomatic factors as contributors, a novel insight emphasizing the complexity of this population.

Therefore, our findings support that children presenting with suspected hereditary connective tissue disorders, characterized by joint hypermobility combined with dysautonomia or psychological symptoms, should be thoroughly evaluated and considered within the EDS spectrum.

According to the 2017 criteria, 25 patients in our cohort were classified within the hypermobility spectrum, where 12 met the full criteria for hEDS and 13 were diagnosed with HSD. This distribution underscores a known limitation of the 2017 classification which is its strict diagnostic thresholds, which are not met by many symptomatic pediatric patients.

The recently proposed 2023 classification offers a more dynamic and developmentally sensitive framework tailored to the pediatric population. Although it was not applied in this study, it would be of interest to evaluate its clinical performance in future cohorts and compare it with the current 2017 standard.

The use of a targeted approach not only reduces unnecessary interventions and genetic testing for those with physiological hypermobility but also facilitates appropriate referrals to multidisciplinary teams for patients at risk of developing systemic manifestations. Ultimately, this leads to improved management strategies, better allocation of healthcare resources, and enhanced quality of life for patients within this spectrum.

In this regard, when evaluating a child who presents with isolated GJH but no other comorbidities, we recommend avoiding applying a diagnostic label such as hEDS, and instead offering reassurance, providing joint protection advice, and appropriate routine physical activity, as well as periodic re-evaluation during follow-up if symptoms persist or develop.

**Figure 2:**
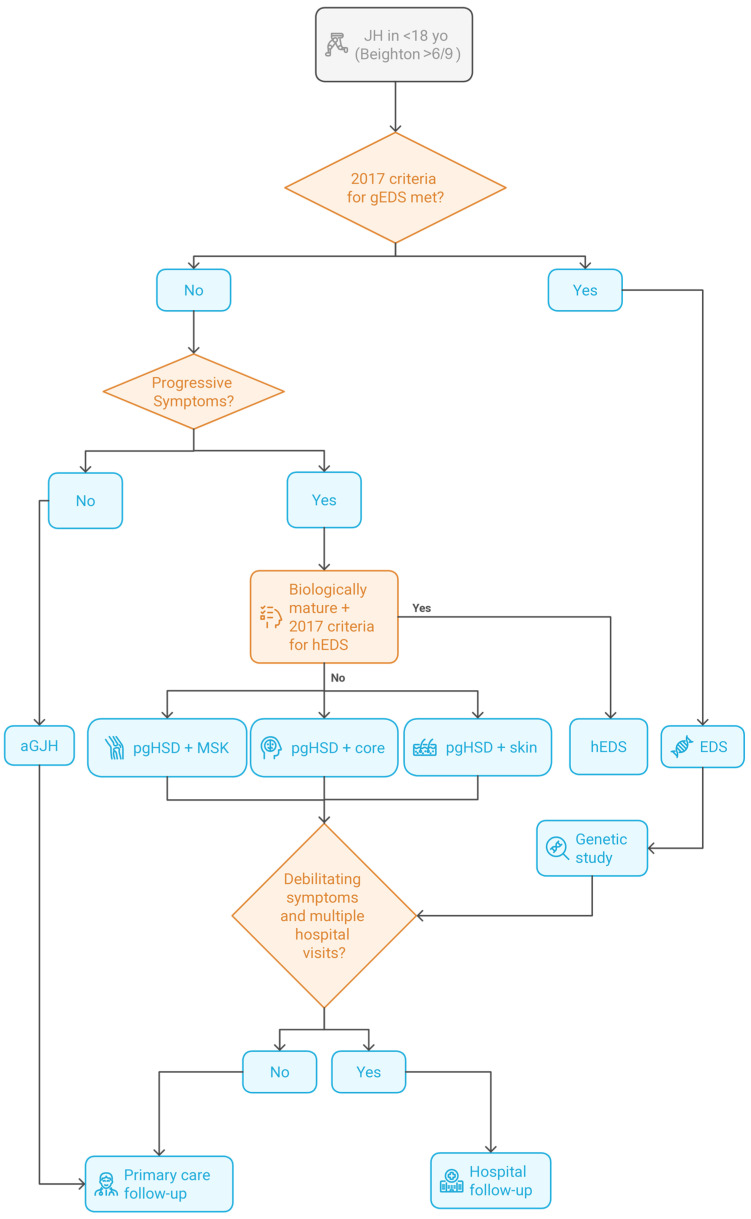
Conceptual diagnostic algorithm in pediatric joint hypermobility and suspected EDS. This flowchart is presented as a theoretical model, based on clinical expertise and studies, and incorporates elements from both the 2017 classification and more recent pediatric frameworks (2023). It suggests appropriate follow-up pathways according to patient phenotype and clinical complexity. The term gEDS refers to all genetically confirmed types of EDS, excluding hEDS, which lacks a known genetic cause. Other terms used, included in the more recent 2023 pediatric classification [3], are as follows: pgHSD = Pediatric Generalized Hypermobility Spectrum Disorder; pgHSD–MSK = pgHSD with musculoskeletal manifestations; pgHSD–core = pgHSD with core comorbidities; pgHSD–skin = pgHSD with cutaneous involvement; and aGJH = asymptomatic generalized joint hypermobility. Genetic study involves clinical evaluation and genetic testing through clinical exome sequencing targeting genes implicated in EDS. Patients with debilitating symptoms and multiple hospital visits, require hospital multidisciplinary follow-up with several specialists within the same year. For an example of a multidisciplinary follow-up proposal, see Figure 3.

**Figure 3:**
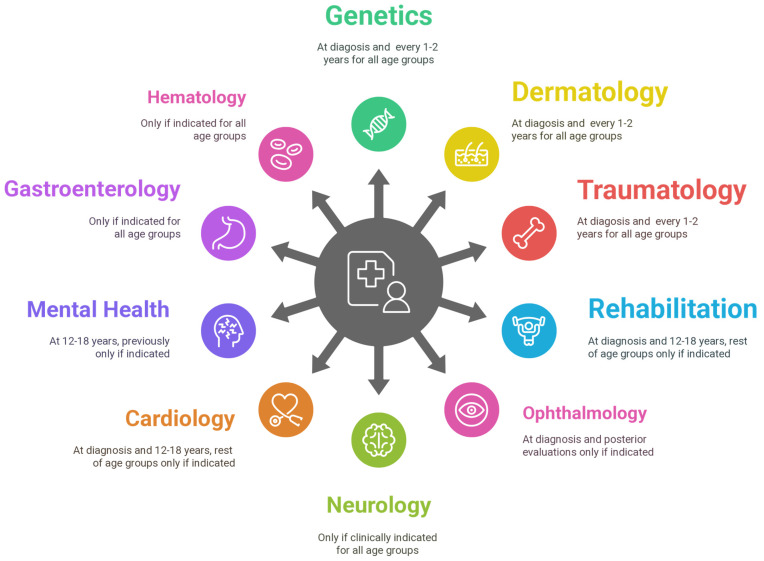
Multidisciplinary follow-up proposal of patients with EDS who experience significant chronic comorbidities requiring frequent hospital care. This diagram proposes a general follow-up approach for patients requiring multiple hospital visits, according to clinical manifestations and EDS subtype. The font size of each specialty in the figure reflects its relative importance in the multidisciplinary follow-up. The main specialties include Medical Genetics, Rehabilitation, Traumatology, and Dermatology, although in some centers, the role of the geneticist may be fulfilled by rheumatology or general pediatrics. It is important to note that vascular and cardiac–valvular EDS require close cardiology monitoring, while classical and dermatosparaxis EDS benefit from dedicated dermatologic care. Children with core comorbidities (e.g., mental health disorders, gastrointestinal issues, skin involvement) or significant musculoskeletal complications benefit from coordinated care with the relevant specialties, including rehabilitation and traumatology. Geneticists frequently coordinate both diagnosis and initial management, especially in cases with chronic joint pain or musculoskeletal complications. Beyond diagnostic tasks, the genetics team often initiates key management strategies, including joint protection advice, recommendations on physical activity, and early referral to physiotherapy. Physical therapy plays a central role in managing joint pain, improving function, and preventing injury through individualized rehabilitation programs focused on joint stability and strengthening. Sports advice is essential, particularly for children involved in repetitive or high-impact activities, to minimize long-term pain and reduce injury risk. Chronic pain is managed using both pharmacologic and non-pharmacologic approaches, prioritizing functional improvement and minimizing unnecessary medication use whenever possible.

Given the broad clinical variability and multisystem involvement observed across different ages, especially in patients with EDS who experience significant chronic comorbidities requiring frequent hospital care, we advocate for a multidisciplinary evaluation approach. In line with this, we include a proposed algorithm (Figure 2) to illustrate a conceptual framework for diagnosis and follow-up in pediatric joint hypermobility. Although it includes elements of both the 2017 and 2023 classifications, it is important to note this tool was not applied yet in our cohort and would be interesting to consider in the future to guide diagnosis and initial clinical classification. In addition to this we have also included a proposed pediatric follow-up protocol (Figure 3) that emphasizes age-specific assessments to optimize ongoing care.

At diagnosis: An initial evaluation by specific subspecialty is performed when the diagnosis is made. If indicated: If deemed necessary by clinicians, the evaluation is conducted based on initial manifestations, compatible symptomatology, and anticipated clinical progression.

Regarding molecular findings, we confirmed the classical, vascular, kyphoscoliotic, and arthrocalasia EDS types genetically, identifying typical mutations described in the literature [1,4,5,15]. As known previously, no definitive molecular marker for hEDS was found, although several VUS in genes such as *TNXB*, *ELN*, *PIEZO2*, and *MYH11* were detected in some patients. All variants were inherited from one of the parents, most of them presenting with similar clinical features. Longitudinal tracking of these patients is planned and future functional studies are also required to determine the pathogenicity of genetic VUS in hEDS. The molecular confirmation of hEDS requires further future studies, as no definitive molecular marker has yet been identified and the variants of uncertain significance (VUS) observed to date require functional validation.

Although our study provides valuable insights, it also has some limitations. First, the cohort size was relatively small, and some data was obtained retrospectively, potentially leading to incomplete information. Second, while genetic testing was performed in a substantial proportion of patients, allowing us to exclude alternative genetic causes of hypermobility such as Marfan and Loeys-Dietz syndromes, it was not systematically performed, particularly among those classified as hEDS/HSD with milder presentations and without familial history. This highlights the need for careful interpretation and future studies with a larger sample and stricter inclusion criteria. It is also important to note that the distribution of EDS subtypes in our cohort is influenced by the nature of referrals to a tertiary Medical Genetics Department, which often include patients with previously identified pathogenic variants in relatives. This process leads to an over-representation of genetically defined subtypes

In conclusion, this study provides comprehensive clinical and molecular insights into pediatric EDS and HSD, highlighting the need for early, multidisciplinary management and proactive screening to detect potential complications. Our findings support the implementation of a pediatric follow-up protocol aimed at optimizing patient care and improving long-term outcomes. Additionally, we establish novel clinical and molecular correlations and emphasize the need for further research into reliable biomarkers for hEDS and HSD.

## Figures and Tables

**Table 1:** Frequency of EDS subtypes in our cohort.

EDS Subtype (2017 Classification)	Frequency (%)
Hypermobile	25 (61)
Classical	9 (22)
Vascular	3 (7.3)
Arthrochalasia	2 (4.9)
Dermatosparaxis	1 (2.4)
Kyphoscoliotic	1 (2.4)

**Table 2:** Epidemiological and clinical characteristics of our cohort.

Category/EDS Subtype	HSD (n = 13)	Hyper-Mobile (n = 12)	Classical (n = 9)	Vascular (n = 3)	Arthrochalasia (n = 2)	Dermatos-Paraxis (n = 1)	Kyphosco-liotic (n = 1)
Demographic Characteristics							
Female	6	4	5	2	1	0	1
Male	7	8	4	1	1	1	0
Mean Age (Years)	8.7	15.1	10.2	10	8	8	14
Initial Evaluation							
Genetic Study Performed	6	8	9	3	2	1	1
Joint Hypermobility	13	12	9	3	1	1	1
Mean Beighton Score	7.3	6.8	8.1	5	9	8.3	8
Traumatological							
Chronic Pain	8	8	3	3	1	1	0
Sprains	5	5	3	2	1	0	0
Fractures	0	0	1	0	0	0	0
Dislocations	6	2	2	0	1	0	0
Cutaneous							
Hyperextensibility	4	3	7	1	1	1	0
Atrophic Scars	1	0	8	1	1	1	1
Hematomas	2	3	7	3	1	1	0
Mental Health							
Anxiety/Depression	1	6	1	1	0	0	0
ASD/Asperger	2	2	1	1	0	0	0
ADHD	1	3	3	0	0	0	0
Chronic Fatigue	2	1	0	0	0	0	0
Cardiovascular							
Aortic Root Dilatation	0	0	0	0	0	0	0
Mitral Valve Prolapse	0	3	0	0	0	0	0
Mitral Regurgitation	0	1	1	0	0	0	0
Postural Orthostatic Tachycardia	0	1	0	0	0	0	0
Syncope	1	5	1	0	0	0	0
Neurological							
Headache	3	5	3	0	0	0	0
Arnold-Chiari Malformation	0	0	0	0	0	0	0
Learning Disabilities	3	2	2	1	0	0	0
Ophthalmological							
Refractive Errors	5	4	6	1	0	0	0
Gastrointestinal							
Chronic Abdominal Pain	3	2	3	0	0	0	0
Food Intolerances/ Allergies	5	0	0	0	0	0	0
Hernias	3	0	3	0	1	0	0

**Table 3:** EDS subtype and molecular findings.

Likely Pathologic (LP) and Pathologic (P) Variants in EDS								
Gene	EDS Subtype	Transcription	Protein Mutation	Nucleotide Mutation	Classification	Genotype	Inheritance	Comment
*COL5A1*	Classic	No data	p.Ser1678Valfs*	c.5031dup	LP ^1^	Htz	DN	N
*COL5A1*	Classic	No data	p.S1678fs*7	c.2331+1G>A	LP	Htz	DN	R
*COL5A1*	Classic	No data	p.Gly610Serfs*	c.1826_1827del	LP	Htz	DN	N
*COL5A1*	Classic	NM_000093.4	-	c.2899-1G>A	LP	Htz	Mother	R
*COL5A1*	Classic	NM_000093.4	p.Tyr1670*	c.5010C>G	P	Htz	DN	N
*COL5A1*	Classic	NM_001278074.1	p.Glu863Val	c.2588A>T	LP	Htz	DN	N
*COL5A1*	Classic	NM_001278074.1	p.Val1140Met	c.3418G>A	LP	Htz	DN	N
*COL5A2*	Classic	NM_000393.3	p.Gly495Asp	c.1484G>A	LP	Htz	DN	N
*COL3A1*	Vascular	NM_000090.3	p.Glu1330Lys	c.3988G>A	LP	Htz	DN	N
*SMAD3*	Vascular	NM_005902.3	p.Arg243Cys	c.727C>T	LP	Htz	DN	R
*COL3A1*	Vascular	NM_000090.3	-	c.1662+1G>A	P	Htz	DN	R
*COL1A2*	Arthrocha-lasia	NM_00089	p.Thr501Ser	c.1502C>G	LP	Htz	DN	N
*COL1A1*	Arthrocha-lasia	NM_00848	p.Pro716Leu	c.2147C>T	LP	Htz	DN	N
*ADAMTS2*	Dermatos-paraxis	NM_014244.4	p.Leu124_Arg125del p.Pro46fs	c.371_376delTGCGGC/ c.137del	LP P	Htz Htz	Mother Father	N R
*PLOD1*	Kyphosco-liotic	NM_001316320.1	p.Arg252_Ile253insCysArg	c.750_755dupCTGCCG/ c.1238+1G>A	LP P	Htz Htz	Mother Father	N R

^1^ LP: likely pathogenic; P: pathogenic; Htz: heterozygous; DN: de novo; N: novel; R: recurrent.

## Data Availability

The raw data supporting the conclusions of this article will be made available by the authors, without undue reservation.

## Supplementary Materials

The following supporting information can be downloaded at https://www.mdpi.com/article/10.3390/genes16080925/s1. Table S1: Variants of Unknown Significance in Patients with hEDS.

## Abbreviations

The following abbreviations are used in this manuscript:

EDS	Ehlers-Danlos Syndrome
hEDS	Hypermobile Ehlers-Danlos Syndrome
GJH	Generalized Joint Hypermobility
HSD	Hypermobility Spectrum Disorders
pgHSD	Pediatric Generalized Hypermobility Spectrum Disorders
