# Supplementary material for: Unveiling the Spectrum: Clinical and Molecular Insights from a Spanish Pediatric Cohort with Hypermobility Disorders and Ehlers-Danlos Syndrome
Source: Genes (Basel). 2025 Jul 31;16(8):925. doi: 10.3390/genes16080925 (PMC12386060; doi:10.3390/genes16080925)
Supplement: Supplementary file 1 [file genes-16-00925-s001.zip › genes-3794884-supplementary.pdf]

genes

MDPI

Supplementary Table 1. Variants of Unknown Significance in patients with hEDS.

Variants of Unknown Significance (VUS) in hEDS						
**Gen**	**Transcription**	**Mutation**	**Classification**	**Genotype**	**Inheritance**	**Subtype**
PIEZO2	NM_022068.3	p.Arg2098Cys/c.6292C>T	VUS	Heterozygosis	Asymptomatic Mother	Hypermobile
TNXB	NM_019105.6	p.Arg724Cys/c.2170C>T	VUS	Heterozygosis	Symptomatic Mother	Hypermobile
MYH11	NM_001040114.1	p.Arg676Cys/c.2026C>T	VUS	Heterozygosis	Symptomatic Father	Hypermobile
ELN	NM_001278939.1	p.Gly410Arg/c.1228G>A	VUS	Heterozygosis	Symptomatic Mother	Hypermobile
TNXB	NM_019105.6	p.Ala1571Asp/c.4712C>A	VUS	Heterozygosis	Symptomatic Mother	Hypermobile
COL6A3	NM_004369.3	p.Arg659Cys/c.1975C>T	VUS	Heterozygosis	NA	Classic
